# Observational study of the coverage rate and survival analysis of COVID-19 vaccination among Indigenous people in Roraima, 2021-2023

**DOI:** 10.1590/S2237-96222025v34e20240762.en

**Published:** 2025-10-24

**Authors:** Daniela da Silva Santos, Paulo Victor de Sousa Viana, Paulo Cesar Basta

**Affiliations:** 1 Escola Nacional de Saúde Pública Sérgio Arouca, Fiocruz, Rio de Janeiro, RJ, Brasil; 2 Centro de Referência Professor Hélio Fraga, Fiocruz, Rio de Janeiro, RJ, Brasil

**Keywords:** Vaccination, Indigenous Peoples, COVID-19, Survival Analysis, Epidemiology, Vacunación, Pueblos Indígenas, COVID-19, Análisis de Supervivencia, Epidemiología

## Abstract

**Objective:**

To estimate vaccination coverage against COVID-19, analyzing survival rates and the delay in vaccination among three Indigenous groups in Roraima between 2021 and 2023.

**Methods:**

This observacional retrospective study is based on vaccination records of urban Indigenous people and Indigenous people assisted by the Eastern Roraima and Yanomami Special Indigenous Health Districts (DSEI), available on the National Health Data Network. Survival and delayed vaccination analyses were conducted by means of the chi-square (χ²), Kaplan-Meier, Cramér’s V, and log-rank tests.

**Results:**

Data from 53,613 vaccinated Indigenous people were analyzed. The average time to complete the vaccination schedule was lower among the Indigenous people served by Eastern Roraima DSEI, which also had a lower rate of delayed vaccination (55.1%). The comparison of the survival curves revealed significant differences between the groups (χ²=1.308; p-value<0.001), and the pairwise analyses confirmed statistical distinctions between all the groups (p-value<0.001).

**Conclusion:**

The study identified low vaccination coverage and significant differences in vaccination rates and patterns between the groups analyzed. The Eastern Roraima DSEI had the best vaccination indicators, with the shortest time to complete the vaccination schedule and the lowest immunization delay.

## Resumen

**Objetivo:**

Estimar la cobertura de vacunación contra la COVID-19, analizando las tasas de supervivencia y el retraso en la vacunación entre tres grupos indígenas de Roraima entre 2021 y 2023.

**Métodos:**

Estudio observacional y transversal basado en registros vacunales de indígenas urbanos y de indígenas atendidos por los Distritos Sanitarios Especiales Indígenas (DSEI) Este Roraima y Yanomami, disponibles en la Red Nacional de Datos en Salud. Se realizaron análisis de supervivencia y retraso en la vacunación utilizando las pruebas de chi-cuadrado (χ²), Kaplan-Meier, coeficiente V de Cramér y log-rank.

**Resultados:**

Se analizaron datos de 53.613 indígenas vacunados. El tiempo promedio para completar el esquema de vacunación fue menor entre los indígenas atendidos por el DSEI Este Roraima, que también presentó una menor tasa de retraso en la vacunación (55,1%). La comparación de las curvas de supervivencia reveló diferencias significativas entre los grupos (χ²=1,308; valor p<0,001), y los análisis por pares confirmaron distinciones estadísticas entre todos los grupos (valor p<0,001).

**Conclusión:**

El estudio identificó una baja cobertura vacunal y diferencias significativas en las tasas y patrones de vacunación entre los grupos analizados. El DSEI Este Roraima presentó los mejores indicadores de vacunación, con menor tiempo para completar el esquema vacunal y menor retraso en la inmunización.

**Ethical aspects**

This research respected ethical principles, having obtained the following approval data:

Research Ethics Committee: Universidade Federal de Roraima

Opinion number: 6.728.592

Approval date: 27/3/2024

Certificate of Submission for Ethical Appraisal: 76405323.4.0000.5302

Informed Consent Form: Exempt

## Introduction

Indigenous people are among the most vulnerable populations in the world, with a life expectancy 20 years lower than that of the non-Indigenous population. This scenario reflects social stigmatization, marginalization, and limited access to public services, which contribute to greater vulnerability to neglected diseases ^1^ ^,^ ^2^.

In many countries, Indigenous people face difficulties in accessing health services due to their remote location and the lack of effective public policies ^3^. In Brazil, this vulnerability has historical roots, dating back to the colonial period, when the occupation of the territory resulted in the invasion of Indigenous lands, population reduction, and the subjugation of native cultures ^4^.

The COVID-19 pandemic has highlighted these inequalities, increasing the risks of illness and mortality among Indigenous people ^5^. Despite efforts to develop and distribute vaccines, vaccination coverage in this population was low - 20 percentage points (pp) lower than in the non-Indigenous population ^6^ ^,^ ^7^. However, this disparity did not occur evenly across the country, and was more pronounced among Indigenous people living in the Legal Amazon. These challenges have significantly impacted vaccination coverage rates in Roraima, one of the states with the largest Indigenous population ^8^ ^,^ ^9^.

In the urban context, Indigenous people face the effects of contact with a non-Indigenous society, which can result in prejudices that compromise health care and quality of life ^10^ ^,^ ^11^. Furthermore, the scarcity of research on this growing population represents a significant gap in the scientific literature ^12^.

Given that monitoring immunization indicators is essential for achieving targets and that at-risk groups have different life contexts that directly influence their health progress, this study aims to estimate vaccination coverage against COVID-19 and analyze survival among three Indigenous groups in Roraima between 2021 and 2023.

## Methods

Design

This is an observational retrospective, and study that analyzes survival and delayed vaccination among three Indigenous groups in Roraima: (i) Indigenous people served by the Eastern Roraima Special Indigenous Health District (DSEI); (ii) Yanomami DSEI; and (iii) those living in urban areas. The period covered is from 2021 to 2023.

Setting

Roraima has an area of 223,644.530 km² and a population of 636,303 inhabitants, of which 15.0% are Indigenous people. 73.0% live on 32 demarcated and ratified Indigenous territories ^13^ ^,^ ^14^.

The health care delivery for Indigenous communities is the responsibility of the Yanomami and Eastern Roraima DSEI, part of the Indigenous Health Subsystem. On the other hand, Indigenous people living in cities have access to the services of the Brazilian National Health System (SUS) ^14^ ^,^ ^15^.

Data collection took place in April 2024, at a single moment, after approval by the ethics committee. Records from the years 2021 to 2023 were included.

Participants

The study included records of Indigenous people aged 6 months and over, of both sexes, who had received at least one dose of the COVID-19 vaccine, registered in the “Indigenous groups and peoples” category in the database.

Records of Indigenous people who received vaccine doses in more than one state (except Yanomamis vaccinated in Amazonas and Roraima), those vaccinated in different locations (different cities, DSEI or urban areas), as well as records with incomplete or inconsistent information regarding data, batch, type of vaccine or social data were excluded.

Variables

The data was analyzed based on the following variables:

Doses administered: Period between 2021 and 2023, type of vaccine (Pfizer, pediatric Pfizer, AstraZeneca, CoronaVac, Janssen, and Pfizer Baby), and number of doses received (1st, 2nd dose, and up to 3rd dose for Pfizer Baby).

Indigenous groups: Yanomami, urban, and Eastern DSEI.

Age group: 0-9 years old, 10-19 years old, 20-59 years old, and ≥60 years old.

Sex: Female and male.

To minimize possible biases, the age variable was considered a possible confounding factor, and therefore, the data were stratified by age group. In addition, the influence of the vaccination site was tested by comparing models with and without the control of this variable, to see if it interfered with the relationship between other variables.

Data source and measurement

The data was obtained from a public and freely accessible source, made available by the Brazilian National Health System Information Technology Department (DATASUS), accessible at: OpenDataSUS. A survey of the total number of records was conducted, followed by applying inclusion and exclusion criteria for statistical analysis.

Bias control

Only records of doses administered to the same patient at the same location were included to minimize information bias, excluding incomplete or inconsistent data. In addition, standardized criteria were adopted for defining the vaccination interval, guaranteeing the validation of the data, and considering that the database is frequently updated and comes from reliable sources.

In order to mitigate the risk of confounding and selection bias, potential confounding factors were identified and controlled, and the inclusion of as many records as possible from the initial target population was considered. To avoid time bias, the data for the entire period from 2021 to 2023 was analyzed together.

Study size

All available records that met the inclusion criteria, excluding those with inconsistencies or incoherencies, were analyzed.

Data availability

The data used in the study is available on the Figshare platform, accessible via the link: Figshare Dataset ^16^.

Statistical methods

The reference vaccination schedule was the one established by the Ministry of Health ^17^ in the years studied, considering the administration of two doses for any vaccine to be a complete schedule, except for Pfizer Baby, which requires three doses.

Delayed vaccination was defined as doses administered five days or more after the recommended interval. The vaccination schedules were:

4 weeks: CoronaVac and Pfizer Baby.

8 weeks: Pediatric Pfizer, Pfizer, AstraZeneca, and Janssen.

The statistical analysis was divided into two stages:

Analysis of the delayed vaccination: calculation of which group had the longest delay in completing the vaccination schedule. 

Completion of vaccination within the ideal timeframe: identifying which group completed vaccination within the timeframe recommended by the Ministry of Health.

In the first stage, survival curves were estimated using the Kaplan-Meier method, considering how far the vaccination schedule had progressed, and compared using the log-rank test. A survival table was also created to assess the proportion of individuals still under observation, i.e., who had neither completed the vaccination schedule nor been excluded.

For statistically significant results, the adjusted standardized residuals (Pearson residuals) were calculated to compare the observed and expected frequencies. Values outside the range [-1.96; 1.96] were considered statistically significant.

In the second stage, the association between delayed vaccination and group was tested using the chi-square test. To measure the strength of this association, Cramér’s V coefficient was used, considering degrees of freedom equal to 1, with the association classified according to the following values:

Small: V≥0.1

Average: V≥0.3

High: V≥0.5 (18)

To handle missing data, all incomplete or inaccurate gaps were excluded.

The data was tabulated and analyzed using R software (version 4.3.3), with a significance level of 5%.

## Results 

For the study, 53,613 records were eligible and effectively analyzed. The majority of those vaccinated (84.1%) were Indigenous people living in rural areas (n=45,094), especially those belonging to the Eastern Roraima DSEI, which represents 60.0% of rural Indigenous people (n=32,183), followed by Indigenous people from the Yanomami DSEI (n=12,938; 39.9%). Urban Indigenous people represent 15.8% (n=8,492) of all vaccinated individuals. In some cities, fewer than 10 Indigenous people were vaccinated during the study period.

The overall vaccination coverage of Indigenous people in Roraima was 40.3%. Specifically, coverage was 30.6% for the Yanomami DSEI, 42.6% for the Eastern Roraima DSEI, and 20.71% for Indigenous people living in urban areas.

Vaccines were most frequently administered to Indigenous people aged 20 to 59 (54.5%), followed by adolescents aged 10 to 19 (25.7%), aged individuals over 60 (12.5%), and children under 10 (7.1%). Vaccination coverage was similar between men and women, although men represented 50.1% of all individuals vaccinated.

In the three groups analyzed, Indigenous people aged 20 to 59 were the most vaccinated, with proportions of 60.4%, 49.0% and 55.1% for urban Indigenous people, the Yanomami DSEI, and Eastern Roraima DSEI, respectively. The most widely used vaccine among the Indigenous people served by the DSEI was Coronavac (70.4% in the Eastern Roraima DSEI and 62.7% in the Yanomami DSEI). Among Indigenous people living in urban areas, the most commonly administered vaccine was Pfizer (65.0%) (Table 1).

Of the 53,613 Indigenous people who received the first dose of the vaccine, only 34,521 (64.4%) completed the vaccination schedule with the second dose, indicating that 35.6% (n=19,092) did not complete the vaccination. On the other hand, 35 Indigenous people received up to six doses of the vaccine.

The survival analysis showed that the Indigenous people from the Eastern Roraima DSEI had the best rates of completion of the vaccination program (47.5%), followed by the Indigenous people from the urban context (35.6%) and those from the Yanomami DSEI (29.4%). The average time to complete the vaccination schedule was lower in the Eastern DSEI, with 35.0% of those vaccinated completing the schedule within 67 days, followed by 373 days for urban Indigenous people and 965 days for Yanomamis, as estimated by the Kaplan-Meier method.

**Table 1:** Sociodemographic characteristics and types of vaccines offered, by Indigenous group. Roraima, 2021 to 2023 (n=53,613)

**Variables**	**Yanomami**	**Eastern Roraima**	**Urban**	**p-value**
	n (%)	**n (%)**	**n (%)**	
**Sex**				<0.001
Female	6,477 (50.0)	15,716 (48.8)	4,508 (53.0)	
Male	6,465 (49.9)	16,463 (51.1)	3,984 (46.9)	
**Age group (years old)**				<0.001
0-9	2,364 (18.2)	3,504 (10.8)	840 (9.8)	
10-19	3,463 (26.7)	8,235 (25.5)	2,121 (24.9)	
20-59	6,351 (49.0)	17,734 (55.1)	5,150 (60.4)	
≥60	761 (5.8)	2,706 (8.4)	403 (4.7)	
**Vaccines**				<0.001
Coronavac	15,462 (62.7)	44,644 (70.4)	2,186 (14.5)	
Pfizer	7,689 (31.1)	18,253 (28.8)	9,810 (65.0)	
Pfizer Baby	840 (3.4)	26 (0.0)	91 (0.6)	
Astrazeneca	660 (2.6)	12 (0.0)	2,709 (17.9)	
Janssen	1 (0.0)	417 (0.6)	277 (1.8)	

**Figure 1.**
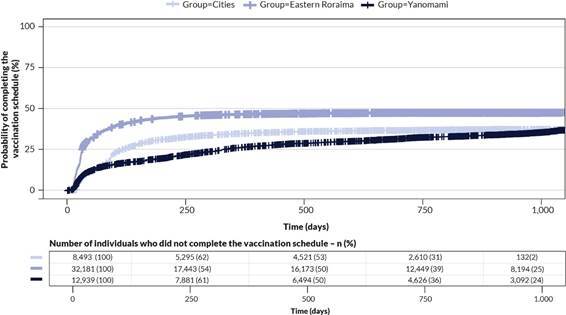
Survival curves of the probability of completing the vaccination schedule over time (in days), by Indigenous group. Roraima, 2021-2023 (n=53,613)

Figure 1 shows that the Indigenous people of the Eastern Roraima DSEI had a vaccination completion rate close to 50.0%, with the curves stabilizing 250 days after the first dose was administered. The log-rank test revealed statistically significant differences between the curves of the three groups analyzed (χ²=1.308; p-value<0.001). The paired comparisons indicated that all the groups differed significantly (p-value<0.001).

Concerning delayed vaccination, the Indigenous people of the Eastern Roraima DSEI had the lowest rate of delayed vaccination (55.1%), compared to the Indigenous people of the Yanomami DSEI (73.0%) and those in an urban context (79.2%) (Table 2).

**Table 2:** Absolute and relative frequencies of vaccinated people with delayed vaccination, by Indigenous group. Roraima, 2021 to 2023 (n=53,613)

**Variables**	**Urban** **(n=8,493)**	**Eastern Roraima** **(n=32,181)**	**Yanomami** **(n=12,939)**	**p-value**	**Cramér’s V**
Delayed Vaccination	**n (%)**	**n (%)**	**n (%)**	**<0.001**	**0.209**
No	1,763 (20.7)	14,427 (44.8)	3,517 (27.1)		
Yes	6,730 (79.2)	17,754 (55.1)	9,422 (72.8)		

The chi-square test of independence, applied to the same population, revealed a statistically significant association between delayed vaccination and the different groups analyzed (χ²=2.347; p-value<0.001; V=0.209). Cramér’s V coefficient indicated a weak association.

The adjusted standardized residuals confirmed that the three groups differed significantly, with the proportion of delayed vaccination among the Indigenous people in the urban context being statistically higher than in the groups from the Yanomami DSEI and Eastern Roraima DSEI.

## Discussion 

In this study, although the Indigenous people from the Eastern Roraima DSEI showed better vaccination results than the other groups, they all had inadequate survival and vaccination timing, considering the recommended interval between the first and second doses. This scenario reflects the challenges of vaccination campaigns, even in contexts of health emergencies, highlighting the need for more effective actions.

The limitations of this research include the use of secondary data, the absence of some clinical information from the population, and the tendency of Cramér’s V to generate relatively low correlations, even for statistically significant results.

The Northern region recorded the worst vaccination coverage rates in the country during the COVID-19 vaccination campaign. Roraima had the lowest vaccination coverage rates among the states in the region ^18^ ^-^ ^20^. Similarly, Indigenous people also recorded some of the lowest vaccination coverage in Brazil, with less than half of eligible individuals vaccinated (48.7%). This result is 30 percentage points lower than observed in the non-Indigenous population, especially in the North, where the complete vaccination schedule reached only 40.3% of Indigenous people ^7^. This study, therefore, corroborates these findings.

Low vaccination coverage among children follows a pattern identified in studies on pediatric vaccination, since children have lower vaccination rates than adults. A study conducted by Fiocruz ^21^ indicated that vaccination against COVID-19 among children in the general population was below the rates of the adult population, which is worrying considering that adult adherence was already lower than expected.

Although aged individuals were a priority group for vaccination, the highest vaccination coverage rates were recorded among Indigenous people aged between 20 and 59, indicating greater adherence to vaccination among the working-age population, possibly due to greater mobility, proficiency in Portuguese, and more frequent exposure to urban centers.

A study ^7^ reported that 65.0% of Indigenous people had partial vaccination coverage in Brazil. In Roraima, only 64.0% adhered to the second dose, indicating a high rate of vaccination schedule dropout.

Considering vaccination schedule completeness within the range recommended by the Ministry of Health, the Indigenous people of the Yanomami DSEI took longer to complete the vaccination schedule compared to the Indigenous people of the Eastern Roraima DSEI. This delay reflects logistical difficulties, access to the communities, and the inconvenience caused by the closure of some health centers in the Yanomami Indigenous territory, resulting from conflicts in mining areas that have already been discussed and investigated in previous research ^3^ ^,^ ^22^ ^-^ ^24^.

In the survival analysis, the Indigenous people from the Eastern Roraima DSEI showed better rates, reflecting the adoption of more effective strategies during the campaign and better organization of assistance. In addition, the Yanomami Indigenous people have logistical difficulties that significantly impact health outcomes, and there is a need to discuss more efficient strategies.

The low vaccination coverage rates also observed among Indigenous people in urban contexts reflect the challenges faced by this group in accessing health services. Their arrival in the cities often exposes them to stigma and discrimination, factors that contribute to their marginalization and make it difficult for them to access the assistance they need ^10^ ^-^ ^11^.

The lack of research into Indigenous people’s social organization and health needs in an urban context represents a significant gap in the scientific literature ^12^. A better understanding of this population is essential for formulating effective public policies and for their equitable reception in health services.

The results highlight the importance of better understanding the needs of these populations, considering their geographical, cultural, and social singularities, and provide input for formulating health policies that reduce ethnic-racial inequalities. Culturally sensitive approaches adapted to local realities are fundamental.

There are still few studies on vaccination coverage among Indigenous people in Brazil. This analysis is expected to be a starting point for expanding the literature, providing more detail on the organization and structure of health services. Building more effective public policies must consider the specificities of these populations and the organization of the health and DSEI systems.
