# Supplementary material for: Observational study of the coverage rate and survival analysis of COVID-19 vaccination among Indigenous people in Roraima, 2021-2023
Source: Epidemiol Serv Saude. 2025 Oct 24;34:e20240762. doi: 10.1590/S2237-96222025v34e20240762.en (PMC12551943; doi:10.1590/S2237-96222025v34e20240762.en)
Supplement: Translation

# Translation: Estudo observacional da taxa de cobertura e análise de sobrevivência da vacinação contra covid-19 entre os indígenas de Roraima, 2021-2023

**DOI:** 10.1590/S2237-96222025v34e20240762.pt

**Authors:** Daniela da Silva Santos, Paulo Victor de Sousa Viana, Paulo Cesar Basta

**Affiliations:** 1 Escola Nacional de Saúde Pública Sérgio Arouca, Fiocruz, Rio de Janeiro, RJ, Brasil; 2 Centro de Referência Professor Hélio Fraga, Fiocruz, Rio de Janeiro, RJ, Brasil

**Keywords:** Vacinação, Povos Indígenas, Covid-19, Análise de Sobrevida, Epidemiologia

## Abstract

**Métodos:**

Estudo observacional retrospectivo baseado em registros vacinais de indígenas urbanos e de indígenas atendidos pelos Distritos Sanitários Especiais Indígenas (DSEI) Leste Roraima e Yanomami, disponíveis na Rede Nacional de Dados em Saúde. Foram realizadas análises de sobrevivência e atraso vacinal por meio de testes qui-quadrado (χ²), Kaplan-Meier, força V de Cramer e log-rank.

**Resultados:**

Foram analisados ​​dados de 53.613 indígenas vacinados. O tempo médio para completar o esquema vacinal foi menor entre os indígenas atendidos pelo DSEI Leste Roraima, que também apresentou menor taxa de atraso vacinal (55,1%). A comparação das curvas de sobrevivência revelou diferenças significativas entre os grupos (χ²=1,308; p-valor<0,001), e as análises entre pares confirmaram distinções estatísticas entre todos os grupos (p-valor<0,001).

**Conclusão:**

O estudo identificou baixa cobertura vacinal e diferenças significativas nas taxas e padrões de vacinação entre os grupos analisados. O DSEI Leste Roraima apresentou os melhores indicadores de vacinação, com menor tempo para completar o esquema vacinal e menor atraso na imunização.

**Aspectos éticos**

Esta pesquisa respeitou os princípios éticos, obtendo os seguintes dados de aprovação:

Comitê de ética em pesquisa: Universidade Federal de Roraima

Número do parecer: 6.728.592

Data de aprovação: 27/3/2024

Certificado de apresentação de apreciação ética: 76405323.4.0000.5302

Registro do consentimento livre e esclarecido: Dispensado

## Introdução

Os indígenas estão entre as populações mais vulneráveis do mundo, com uma expectativa de vida 20 anos inferior à da população não indígena. Esse cenário reflete a estigmatização social, a marginalização e o acesso limitado aos serviços públicos, que contribuem para uma maior vulnerabilidade a doenças negligenciadas ^1^ ^,^ ^2^.

Em muitos países, os indígenas enfrentam dificuldades para acessar serviços de saúde, devido à localização remota e à ausência de políticas públicas eficazes ^3^. No Brasil, essa vulnerabilidade tem raízes históricas, remontando ao período colonial, quando a ocupação do território resultou na invasão de terras indígenas, na redução populacional e na subjugação das culturas nativas ^4^.

A pandemia da covid-19 evidenciou essas desigualdades, ampliando os riscos de adoecimento e mortalidade entre os indígenas ^5^. Apesar dos esforços para o desenvolvimento e distribuição de vacinas, a cobertura vacinal nessa população foi baixa - 20 pontos percentuais (p.p.) inferior à população não indígena ^6^ ^,^ ^7^. No entanto, essa disparidade não ocorreu de forma homogênea no país, sendo mais acentuada entre os indígenas que vivem na Amazônia Legal. Em Roraima, um dos estados com maior população indígena do país, esses desafios impactaram significativamente as taxas de cobertura vacinal ^8^ ^,^ ^9^.

No contexto urbano, os indígenas enfrentam os efeitos do contato com a sociedade não indígena, o que pode resultar em preconceitos que comprometem a assistência à saúde e qualidade de vida ^10^ ^,^ ^11^. Além disso, a escassez de pesquisas sobre essa população em crescimento representa uma lacuna significativa na literatura científica ^12^.

Dado que o monitoramento dos indicadores de imunização é essencial para o alcance das metas e que grupos de risco apresentam diferentes contextos de vida que influenciam diretamente seus avanços de saúde, este estudo pretende estimar a cobertura vacinal contra a covid-19 e analisar a sobrevivência entre três grupos indígenas de Roraima entre 2021 e 2023.

## Métodos 

Delineamento

Este é um estudo observacional retrospectivo que analisa a sobrevivência e o atraso vacinal entre três grupos indígenas de Roraima: i) indígenas atendidos pelo Distrito Sanitário Especial Indígena (DSEI) Leste Roraima; ii) DSEI Yanomami; e (iii) residentes em áreas urbanas. O período trabalhado compreende os anos de 2021 a 2023.

Contexto

Roraima possui uma área de 223.644,530 km² e uma população de 636.303 habitantes, dos quais 15,0% são indígenas. Destes, 73,0% vivem em 32 terras indígenas demarcadas e homologadas ^13^ ^,^ ^14^.

A assistência à saúde dos indígenas aldeados é de responsabilidade dos DSEI Yanomami e Leste Roraima, que integram o Subsistema de Saúde Indígena. Já os indígenas que vivem nas cidades têm acesso aos serviços do Sistema Único de Saúde (SUS) ^14^ ^,^ ^15^.

A coleta dos dados ocorreu em abril de 2024, em um único momento, após aprovação pelo comitê de ética. Foram incluídos registros dos anos de 2021 a 2023.

Participantes

Foram incluídos no estudo registros de indígenas a partir dos 6 meses de idade, de ambos os sexos, que receberam ao menos uma dose da vacina contra a covid-19, cadastrados na categoria “grupo e povos indígenas” no banco de dados.

Foram excluídos registros de indígenas que receberam doses de vacina em mais de um estado (exceto Yanomamis vacinados no Amazonas e em Roraima), aqueles vacinados em locais distintos (cidades diferentes ou entre DSEI e áreas urbanas), bem como registros com informações incompletas ou inconsistentes quanto a dados, lote, tipo de vacina ou dados sociais.

Variáveis

 Os dados foram analisados com base nas seguintes variáveis:

Doses aplicadas: Período entre 2021 e 2023, tipo de vacina (Pfizer, Pfizer pediátrica, AstraZeneca, CoronaVac, Janssen e Pfizer Baby) e número de doses recebidas (1ª, 2ª dose e até 3ª dose para Pfizer Baby).

Grupos indígenas: Yanomami, urbanos e DSEI Leste.

Faixa etária: 0-9 anos, 10-19 anos, 20-59 anos e ≥60 anos.

Sexo: Feminino e masculino.

Para minimizar possíveis vieses, a variável idade foi considerada um possível fator de confusão e, portanto, os dados foram estratificados por faixa etária. Além disso, a influência do local de vacinação foi testada comparando-se modelos com e sem o controle dessa variável, para se verificar se interferia na relação entre outras variáveis.

Fontes de dados e mensuração

Os dados foram obtidos de uma fonte pública e de acesso livre, disponibilizada pelo Departamento de Informática do SUS, acessível em: OpenDataSUS. Foi realizado um levantamento da quantidade total de registros, seguido da aplicação dos critérios de inclusão e exclusão para análise estatística.

Controle de viés

Para minimizar o viés da informação, foram incluídos apenas registros de doses administradas a um mesmo paciente em um mesmo local, excluindo-se dados incompletos ou inconsistentes. Além disso, foram adotados critérios padronizados para definição do intervalo vacinal, garantindo a validação dos dados, considerando-se também que o banco de dados é frequentemente atualizado e proveniente de fontes confiáveis.

Para mitigar riscos de viés de confusão e seleção, foram identificados e controlados fatores potenciais de confusão, além de se considerar a inclusão do maior número possível de registros da população-alvo inicial. Para evitar viés temporal, os dados de todo o período de 2021 a 2023 foram analisados de forma conjunta.

Tamanho do estudo

Todos os registros disponíveis que atendem aos critérios de inclusão foram analisados, com exclusão daqueles com inconsistências ou incoerências.

Disponibilidade dos dados

Os dados utilizados no estudo estão disponíveis na plataforma Figshare, acessíveis pelo link: Figshare Dataset ^16^.

Métodos estatísticos

O esquema vacinal seguido foi o previsto pelo Ministério da Saúde ^17^ nos anos estudados, considerando-se esquema completo a administração de duas doses para qualquer vacina, exceto a Pfizer Baby, que exige três doses.

Atrasos vacinais foram definidos como doses administradas a partir de cinco dias após o intervalo recomendado. Os esquemas vacinais foram:

4 semanas: CoronaVac e Pfizer Baby.

8 semanas: Pfizer pediátrica, Pfizer, AstraZeneca e Janssen.

A análise estatística foi dividida em duas etapas:

Análise do atraso vacinal: calculou-se qual grupo apresentou maior atraso na conclusão do esquema vacinal.

Conclusão da vacinação dentro do prazo ideal: identificou-se qual grupo finalizou a vacinação dentro do intervalo recomendado pelo Ministério da Saúde.

Na primeira etapa, as curvas de sobrevivência foram estimadas pelo método de Kaplan-Meier, considerando-se como avançou a completude do esquema vacinal, e comparados pelo teste log-rank. Também foi construída uma tabela de sobrevivência para avaliar a proporção de indivíduos ainda sob observação, ou seja, que não tinham completado o esquema vacinal nem sido censurados.

Para os resultados estatisticamente significativos, foram calculados os resíduos padronizados ajustados (resíduos de Pearson), a fim de se compararem as frequências observadas e esperadas. Valores fora do intervalo [-1,96; 1,96] foram considerados estatisticamente significativos.

Na segunda etapa, a associação entre atraso vacinal e grupo de pertencimento foi testada por meio do teste qui-quadrado. Para medir a força dessa associação, utilizou-se o coeficiente V de Cramer, considerando-se graus de liberdade iguais a 1, com classificação da associação conforme os seguintes valores:

Pequena: V≥0,1

Média: V≥0,3

Alta: V≥0,5 (18)

Para lidar com dados ausentes, todas as lacunas incompletas ou imprecisas foram excluídas.

A tabulação e a análise dos dados foram realizadas com utilização do software R (versão 4.3.3), adotando-se um nível de significância de 5%.

## Resultados 

Para o estudo, foram elegíveis e efetivamente analisados 53.613 registros. A maioria dos vacinados (84,1%) eram indígenas residentes na zona rural (n=45.094), com destaque para aqueles pertencentes ao DSEI Leste Roraima, que representaram 60,0% dos indígenas da zona rural (n=32.183), seguidos pelos indígenas do DSEI Yanomami (n=12.938; 39,9%). Indígenas em contexto urbano corresponderam a 15,8% (n=8.492) do total de vacinados. Em alguns municípios, menos de 10 indígenas foram vacinados durante o período do estudo.

A cobertura vacinal geral dos indígenas em Roraima foi de 40,3%. Especificamente, a cobertura foi de 30,6% para o DSEI Yanomami, 42,6% para o DSEI Leste Roraima e 20,71% para indígenas residentes em áreas urbanas.

As vacinas foram mais frequentemente administradas em indígenas de 20 a 59 anos (54,5%), seguidos por adolescentes de 10 a 19 anos (25,7%), idosos com mais de 60 anos (12,5%) e crianças com menos de 10 anos (7,1%). A cobertura vacinal foi semelhante entre homens e mulheres, embora os homens tenham representado 50,1% do total de vacinados.

Nos três grupos analisados, os indígenas de 20 a 59 anos foram os mais vacinados, com proporções de 60,4%, 49,0% e 55,1% para indígenas urbanos, DSEI Yanomami e DSEI Leste Roraima, respectivamente. A vacina mais utilizada entre os indígenas atendidos pelos DSEI foi a Coronavac (70,4% no DSEI Leste Roraima e 62,7% no DSEI Yanomami). Já entre os indígenas em contexto urbano, a vacina mais administrada foi a Pfizer (65,0%) (Tabela 1).

Dos 53.613 indígenas que receberam a primeira dose da vacina, apenas 34.521 (64,4%) completaram o esquema vacinal com a segunda dose, indicando que 35,6% (n=19.092) não concluíram a vacinação. Por outro lado, 35 indígenas receberam até seis doses da vacina.

A análise de sobrevivência indicou que os indígenas do DSEI Leste Roraima apresentaram os melhores índices de conclusão do esquema vacinal (47,5%), seguidos pelos indígenas em contexto urbano (35,6%) e os do DSEI Yanomami (29,4%). O tempo médio para completar o esquema vacinal foi menor no DSEI Leste, com 35,0% dos vacinados concluindo o esquema em até 67 dias, seguido de 373 dias para indígenas urbanos e 965 dias para Yanomamis, conforme estimado pelo método de Kaplan-Meier.

**Tabela 1:** Características sociodemográficas e tipos de vacinas ofertadas, por grupo indígena. Roraima, 2021 a 2023 (n=53.613)

**Variáveis**	**Yanomami**	**Leste Roraima**	**Urbanos**	**p-valor**
	n (%)	n (%)	n (%)	
**Sexo**				<0,001
Feminino	6.477 (50,0)	15.716 (48,8)	4.508 (53,0)	
Masculino	6.465 (49,9)	16.463 (51,1)	3.984 (46,9)	
Faixa etária (anos)				<0,001
0-9	2.364 (18,2)	3.504 (10,8)	840 (9,8)	
10-19	3.463 (26,7)	8.235 (25,5)	2.121 (24,9)	
20-59	6.351 (49,0)	17.734 (55,1)	5.150 (60,4)	
≥60	761 (5,8)	2.706 (8,4)	403 (4,7)	
**Vacinas**				<0,001
Coronavac	15.462 (62,7)	44.644 (70,4)	2.186 (14,5)	
Pfizer	7.689 (31,1)	18.253 (28,8)	9.810 (65,0)	
Pfizer Baby	840 (3,4)	26 (0,0)	91 (0,6)	
Astrazeneca	660 (2,6)	12 (0,0)	2.709 (17,9)	
Janssen	1 (0,0)	417 (0,6)	277 (1,8)	

**Figura 1:** Curvas de sobrevivência da probabilidade de completar o esquema vacinal ao longo do tempo (dias), por grupo indígena. Roraima, 2021-2023 (n=53.613)

Na Figura 1, observa-se que os indígenas do DSEI Leste Roraima apresentaram uma taxa de completude vacinal próxima a 50,0%, com estabilização das curvas a partir de 250 dias da administração da primeira dose. O teste log-rank revelou diferenças estatisticamente significativas entre as curvas dos três grupos analisados (χ²=1.308; p-valor<0,001). As comparações pareadas indicaram que todos os grupos diferem significativamente entre si (p-valor<0,001).

Em relação ao atraso vacinal, os indígenas do DSEI Leste Roraima apresentaram a menor taxa de atraso (55,1%), em comparação com os indígenas do DSEI Yanomami (73,0%) e aqueles em contexto urbano (79,2%) (Tabela 2).

**Tabela 2:** Frequências absoluta e relativa de vacinados com atraso vacinal, por grupo indígena. Roraima, 2021 a 2023 (n=53.613)

**Variáveis**	**Urbanos** **(n=8.493)**	**Leste Roraima** **(n=32.181)**	**Yanomami** **(n=12.939)**	**p-valor**	**V de Cramer**
**Atraso vacinal**	**n (%)**	**n (%)**	**n (%)**	**<0,001**	**0,209**
Não	1.763 (20,7)	14.427 (44,8)	3.517 (27,1)		
Sim	6.730 (79,2)	17.754 (55,1)	9.422 (72,8)		

O teste qui-quadrado de independência, aplicado à mesma população, revelou uma associação estatisticamente significativa entre o atraso vacinal e os diferentes grupos analisados (χ²=2.347; p-valor<0,001; V=0,209). O coeficiente V de Cramer indicou uma força de associação pequena.

Os resíduos padronizados ajustados confirmaram que os três grupos diferem significativamente entre si, sendo que a proporção de atrasos entre os indígenas em contexto urbano foi estatisticamente superior às dos grupos do DSEI Yanomami e do DSEI Leste Roraima.

## Discussão 

Neste estudo, embora os indígenas do DSEI Leste Roraima tenham apresentado melhores resultados vacinais em comparação aos demais grupos, todos tiveram sobrevivência e tempo vacinal inadequados, considerando-se o intervalo preconizado entre a primeira e a segunda dose. Esse cenário reflete desafios das campanhas de vacinação, mesmo em contextos de emergência sanitária, evidenciando a necessidade de ações mais efetivas.

Dentre as limitações desta investigação, destacam-se o uso de dados secundários, a ausência de algumas informações clínicas da população e a tendência do V de Cramer a gerar correlações relativamente baixas, mesmo para resultados estatisticamente significativos.

A região Norte registrou os piores índices de cobertura vacinal do país durante a campanha de vacinação contra a covid-19. Roraima apresentou as menores taxas de cobertura vacinal entre os estados da região ^18^ ^-^ ^20^. Analogamente, os indígenas também registraram uma das menores coberturas vacinais no Brasil, com menos da metade dos elegíveis vacinados (48,7%). Esse resultado é 30 p.p. inferior ao observado na população não indígena, especialmente na região Norte, onde o esquema vacinal completo alcançou apenas 40,3% dos indígenas ^7^. Assim, o presente estudo corrobora esses achados.

A baixa cobertura vacinal entre crianças segue um padrão identificado em estudos sobre vacinação pediátrica, uma vez que as crianças apresentaram taxas de vacinação inferiores às dos adultos. Um estudo conduzido pela Fiocruz ^21^ indicou que a vacinação contra a covid-19 entre crianças na população geral ficou abaixo dos índices da população adulta, o que é preocupante, considerando-se que a adesão dos adultos já estava aquém do esperado.

Apesar de os idosos terem sido grupo prioritário para a vacinação, as maiores coberturas vacinais foram registradas entre indígenas de 20 a 59 anos, indicando maior aderência à vacinação em idade produtiva, possivelmente devido à maior mobilidade, ao domínio do português e à exposição mais frequente aos centros urbanos.

Conforme relatado em estudo conduzido por Pescarini et al. ^7^ , 65,0% dos indígenas tiveram cobertura vacinal parcial no Brasil. Em Roraima, apenas 64,0% aderiram à segunda dose, indicando uma taxa elevada de abandono do esquema vacinal.

Considerando-se a completude vacinal dentro do intervalo recomendado pelo Ministério da Saúde, os indígenas do DSEI Yanomami levaram mais tempo para completar o esquema vacinal, em comparação aos indígenas do DSEI Leste Roraima. Esse atraso reflete dificuldades logísticas e de acesso às aldeias, além dos transtornos provocados pelo fechamento de algumas unidades de saúde na terra indígena Yanomami, decorrentes de conflitos em áreas de garimpo já discutidos e investigados em pesquisas anteriores ^3^ ^,^ ^22^ ^-^ ^24^.

Na análise de sobrevivência, os indígenas do DSEI Leste Roraima apresentaram melhores taxas, refletindo a adoção de estratégias mais eficazes durante a campanha e melhor organização da assistência. Além disso, os indígenas Yanomami apresentam dificuldades logísticas que impactam significativamente os resultados de saúde, havendo a necessidade de discutir estratégias mais eficientes.

Os baixos índices de cobertura vacinal também observados entre indígenas em contexto urbano refletem os desafios de acesso desse grupo aos serviços de saúde. A chegada às cidades frequentemente os expõe a estigmas e discriminação, fatores que contribuem para marginalização e dificultam o acesso à assistência necessária ^10^ ^-^ ^11^.

A carência de pesquisas sobre a organização social e as necessidades de saúde dos indígenas em contexto urbano representa uma lacuna significativa na literatura científica ^12^. Compreender melhor essa população é essencial para a formulação de políticas públicas eficazes e para seu acolhimento equitativo nos serviços de saúde.

Nossos resultados destacam a importância de maior compreensão das necessidades dessas populações, considerando-se suas singularidades geográficas, culturais e sociais e fornecem subsídios para a formulação de políticas de saúde que reduzam as desigualdades étnico-raciais. Abordagens culturalmente sensíveis e adaptadas às realidades locais são fundamentais.

Ainda há poucos estudos sobre a cobertura vacinal entre indígenas no Brasil. Espera-se que esta análise seja ponto de partida para a ampliação da literatura, detalhando melhor a organização e a estrutura dos serviços de saúde. A construção de políticas públicas mais efetivas deve considerar as especificidades dessas populações, bem como a organização dos sistemas de saúde e DSEI.
